# The Apoptosis Regulator 14-3-3η and Its Potential as a Therapeutic Target in Pituitary Oncocytoma

**DOI:** 10.3389/fendo.2019.00797

**Published:** 2019-11-28

**Authors:** Sida Zhao, Bin Li, Chuzhong Li, Hua Gao, Yazhou Miao, Yue He, Hongyun Wang, Lei Gong, Dan Li, Yazhuo Zhang, Jie Feng

**Affiliations:** ^1^Cell Biology, Beijing Neurosurgical Institute, Capital Medical University, Beijing, China; ^2^Beijing Tiantan Hospital, Capital Medical University, Beijing, China; ^3^Beijing Institute for Brain Disorders Brain Tumor Center, Capital Medical University, Beijing, China; ^4^China National Clinical Research Center for Neurological Diseases, Beijing, China; ^5^Chinese Medical Association, Beijing, China

**Keywords:** pituitary, adenoma-pathology, 14-3-3η, CoIP coimmunoprecipitation, mass spectrum, proteomics, tumor proliferation

## Abstract

The 14-3-3 protein family has attracted much attention in research into the pathogenesis of human tumors because of its involvement in tumorigenesis. In previous studies, we found that 14-3-3η was highly expressed in pituitary oncocytoma. However, the mechanism by which 14-3-3η regulates tumorigenesis in pituitary oncocytoma is unclear. 14-3-3η-binding proteins were investigated in pituitary oncocytoma by immunoprecipitation and proteomic analysis. A total of 443 proteins were identified as 14-3-3η binding proteins. The interactions of 14-3-3η and its binding partners were identified by a network analysis using the STRING database. The network included 433 nodes and 564 edges. PRAS40 (AKT1S1) was a binding protein of 14-3-3η and showed experimental interactions with 14-3-3η in the STRING database. The combined score was 0.407, which suggested a functional link. The 443 binding proteins of 14-3-3η showed enriched molecular signatures in GSEA and GO analysis. PRAS40 (AKT1S1) was enriched in the mTOR signaling pathway. Western blot analysis showed that the relative expression of p-PRAS40 (T246)/PRAS40 was significantly higher in pituitary oncocytoma than in normal pituitary tissues (*p* < 0.05). R18, a 14-3-3 protein inhibitor, inhibited MMQ cell proliferation after treatment with 8 μM R18 for 48 h compared to the control group (*p* < 0.01). These results suggest that 14-3-3η may be involved in promoting tumorigenesis in pituitary oncocytoma by interacting with PRAS40 (T246) via the mTOR signaling pathway.

## Introduction

Pituitary adenomas are derived from adenohypophyseal cells, and the prevalence of these lesions ranges from 1/865 to 1/2688 (1). Non-functioning pituitary adenomas (NFPAs) account for one-third of all pituitary adenomas (2) and often develop into macroadenomas, making complete surgical resection difficult (3). Meanwhile, no effective drugs exist for the treatment of NFPAs. Therefore, potential therapeutic targets in NFPAs are needed.

Pituitary oncocytomas are a subtype of NFPAs that were first reported by Kovacs in 1973 (4). A pathological examination of pituitary oncocytoma revealed their immuno-negativity for pituitary hormones, and an electron microscopic examination revealed low levels of cytoplasmic organelles, few round secretary granules, and many mitochondria (5).

The 14-3-3 proteins (14-3-3s) are brain-specific acidic proteins (6) expressed in all eukaryotic cells (7). Seven isoforms (β, ϵ, γ, η, σ, τ, and ζ) have been described in this family, and these proteins currently belong to a highly conserved multigene family. These proteins play key roles in regulating intracellular signaling pathways, including cell proliferation, apoptosis, and protein localization (8). In general, the 14-3-3 protein family can be identified by a phosphorylated serine/threonine in the unique sequence motifs “RSXpS/Ptxp” and “RXXXpS/pTXP” (9, 10). For example, 14-3-3 proteins can bind to phosphorylated PRAS40 (a 40 kDa proline-rich Akt substrate), which decreases the levels of proapoptotic proteins and may lead to the inhibition of apoptosis (11).

The 14-3-3 proteins play important roles in cancers. Elevated expression of 14-3-3ζ is associated with cell proliferation and migration of tongue squamous cell carcinoma (12). Overexpression of 14-3-3γ is closely correlated with tumorigenesis in human lung cancer (13). In our previous study, we demonstrated increased expression of isoform 14-3-3η in pituitary oncocytoma at the protein and mRNA levels (14). However, the function of 14-3-3η in the tumorigenesis of pituitary oncocytoma is still unknown. The binding partners of 14-3-3η in pituitary oncocytoma is also unclear. In this study, for the first time, we explored the binding partners of 14-3-3η in pituitary oncocytoma using co-immunoprecipitation (co-IP) and LC-MS/MS. These binding proteins could play important roles in and may provide a potential treatment target for pituitary oncocytoma.

## Subjects and Methods

### Subjects

All samples of null cell adenomas and oncocytomas were obtained from Tiantan Hospital (Beijing, China) during 2013–2015 following transsphenoidal surgery. The classification of null cell adenomas and oncocytomas was based on immunohistochemical staining and ultrastructural electron microscopy. The null cell adenomas and oncocytomas were negative in hormonal pathological immunostaining. Null cell adenoma had few mitochondria and secretory granules and oncocytoma had large amounts of mitochondria under anti-mitochondrial antibody staining and electron microscopy (Supplementary Figure 2). Based on the 2017 WHO classification (15), the oncocytic adenomas that were selected for our study were variants of null cell adenomas. Fresh tumor samples were frozen at −80°C in isopentane and stored in liquid nitrogen. Ten tumor samples were used for Co-IP and LC-MS/MS analysis, and six tumor samples were used for Western blot. Five samples in the two entities were overlapped. The clinicopathological characteristics of these adenomas are listed in Supplementary Table 1. Healthy pituitary glands obtained from three adult males within 12 h of the donors' involvement in fatal accidents were used as controls. Informed consent was obtained from all patients, and the study was approved by the local ethics committees of Beijing Tiantan Hospital (KY2013-015-02) and performed in full compliance with all of the principles of the Declaration of Helsinki.

### Co-IP of 14-3-3-Binding Proteins

Cell lysis was carried out using lysis buffer [20 mM Tris-HCl, pH 7.5; 1% NP-40; 150 mM NaCl; 1 mM EDTA; 5% glycerol; and EDTA-free protease inhibitor cocktail (Roche)]. The homogenate was rotated for 30 min on ice. After centrifugation at 10,000 × g for 30 min at 4°C, the supernatant was collected. Two micrograms of anti-EE antibodies and 30 μL of protein A/G beads were added to the lysates. Samples were incubated on a rotating wheel overnight at 4°C. The beads were collected by centrifugation at 1,000 RPM for 2 min at 4°C and washed five times with ice-cold PBS buffer. Immunocomplex samples were directly boiled in 2 × SDS-PAGE sample buffer (50 mm Tris-HCl, pH 6.8; 2% SDS; 10% glycerol; 1% β-mercaptoethanol; 12.5 mm EDTA; and 0.02% bromophenol blue), followed by SDS-PAGE and Western blot analysis. Gels were stained with Coomassie blue, cut into small pieces, destained using a solution of 50% acetonitrile and 25 mM ammonium bicarbonate buffer, and then dried with 100% acetonitrile. Modified sequencing-grade trypsin (Promega) at a concentration of 10 μg/mL in 25 mM ABC buffer was added; digestion was carried out overnight at 37°C and terminated by the addition of trifluoroacetic acid (TFA) to a final concentration of 0.1% (v/v). Peptides were extracted from the gel twice with 200 μL of 50% acetonitrile and 0.1% TFA for 30 min. The extracted solution was pooled, lyophilized, and stored at −80°C until use.

### Identification of 14-3-3-Binding Proteins by nanoLC-MS/MS

Peptides were analyzed by nanoLC-MS/MS on a Q Exactive mass spectrometer (Thermo Fisher, USA). Chromatography was performed with solvent A (Milli-Q (Millipore, Billerica, MA) water with 2% acetonitrile and 0.1% formic acid) and solvent B (90% acetonitrile with 0.1% formic acid). Peptides were eluted in 5% solvent B at 300 nL/min for 5 min, 5–40% solvent B for 65 min, 40–80% solvent B for 1 min, and 80% solvent B for 5 min before returning to 5% solvent B for 20 min. The Q Exactive instrument was operated in information-dependent data acquisition mode to switch automatically between MS and MS/MS acquisition. MS spectra were acquired across the mass range of 350–2,000 m/z. Automated peak recognition, 10 s dynamic exclusion, and tandem MS of the top 10 most intense precursor ions at 30% normalized collision energy were performed using Xcalibur software (Thermo Scientific). All MS/MS data were analyzed using Mascot (Matrix Science, London, UK; version 2.3.0). Mascot was set up to search the UniPort human database, which contains 20,199 protein sequences. Mascot was searched with a fragmentation mass tolerance of 0.050 Da and a parent ion tolerance of 10.0 ppm. Carbamidomethylation of cysteine residues was specified as a fixed modification. Oxidation of methionine residues was specified in Mascot as a variable modification. Only proteins with a *p* < 0.05 were accepted.

### Network Involving 14-3-3η Binding Proteins

STRING version 10.5 (https://string-db.org) was used to identify the functional protein association network of 14-3-3η and its binding proteins. The network was used to summarize the interactions of 14-3-3η and its binding proteins. The pop-up windows provided information on nodes and edges. The settings were changed to determine the meanings of network edges and their molecular action. A line indicated the predicted mode of each action. The active interaction source was specified as experiments. The network display mode was set to interactive svg, and display simplifications were used to hide disconnected nodes in the network.

### Western Blot Analysis

Protein was extracted from six pituitary oncocytoma and three healthy pituitary gland tissues using a total protein extraction kit (cat. #2140, Millipore, Billerica, MA, USA). Protein concentrations were measured using the BCA protein assay kit (23225, Pierce, Rockford, IL, USA). Soluble proteins (30 μg) were separated by 10% sodium dodecyl sulfate–polyacrylamide gel electrophoresis (SDS-PAGE), transferred to nitrocellulose membranes, and incubated with blocking buffer (5% non-fat milk) in Tris-buffered saline/Tween 20 (TBST) for 1 h at room temperature. Membranes were then probed with the corresponding primary antibody overnight at 4°C followed by three 10-min washes with TBST. Anti-PRAS40 (phospho-T246) (cat. # ab134084, dilution factor 1:2,000), anti-PRAS40 (cat. # ab151719, dilution factor 1:1,000), anti-FOXO3A (phospho-T253) (cat. # ab154786, dilution factor 1:200), anti-FOXO3A (cat. # ab17026, dilution factor 1:500), anti-YAP1 (phospho-S127) (cat. # ab76252, dilution factor 1:2,000), anti-BAD (phospho-S112) (cat. # ab129192, dilution factor 1:1,000), and anti-BAD (cat. # ab32445, dilution factor 1:1,000) antibodies were obtained from Abcam, Inc. (Cambridge, MA, USA). Subsequently, membranes were incubated with horseradish peroxidase-conjugated secondary antibodies at room temperature for 1 h. An enhanced chemiluminescence kit was used according to the manufacturer's instructions (Amersham Pharmacia Biotech, Piscataway, NJ, USA) to visualize positive bands on nitrocellulose membranes following exposure. The final data were subjected to grayscale scanning and semi-quantitative analysis using ImageJ software (Bio-Rad, Hercules, CA, USA).

### Cell Culture and Reagents

MMQ cells were purchased from American Type Culture Collection (ATCC; Manassas, VA, USA) and were cultured in F12K medium (ATCC; Manassas, VA, USA) supplemented with 2.5% fetal bovine serum (FBS; Gibco) and 15% horse medium (Gibco).

### Cell Proliferation Assay

The proliferation of MMQ cells treated with R18 (SML0108, SIGMA) was assessed by a 3-(4,5-dimethylthiazol-2-yl)-5-(3-carboxymethoxyphenyl)-2-(4-sulfophenyl)-2H-tetrazolium (MTS) assay. Cells were plated into 96-well dishes with 10,000 cells and 100 μL of medium per well and incubated overnight. R18 powder was dissolved in MMQ cell line medium. After 24 h of culture, 8 μM of R18 was added to each well. After 24 and 48 h of R18 treatment, 20 μL of MTS was added to each well, and incubation was continued for 3 h. The absorbance of the wells was measured at 490 nm using a microplate reader (Synergy H1, BioTek). Experiments were performed in triplicate.

### Bioinformatic and Statistical Analysis

The 14-3-3-Pred database was used to identify and analyze the 444 interacting partners of 14-3-3η (www.compbio.dundee.ac.uk/1433pred). Functional annotation databases were utilized based on the biological process, molecular function, and cellular component classifications of 14-3-3η binding proteins as determined by Gene Ontology (GO) (available online at www.geneontology.org). The enrichment pathway analysis of 14-3-3η binding proteins was performed based on the Hallmark Gene Sets of Molecular Signatures database (Gene Set Enrichment Analysis, GSEA, http://software.broadinstitute.org/gsea/msigdb/index.jsp).

All statistical analyses were conducted using the GraphPad Prism software package (GraphPad Software, San Diego, CA 92108). Unpaired Student's *t*-tests and chi-squared (Fisher's exact) tests were used for comparisons of quantitative and qualitative data, respectively. Differences with a *p* < 0.05 were considered significant.

## Results

### Binding Partners of 14-3-3η in Acidophil Stem Cell Adenoma

Our previous study showed that the expression level of 14-3-3η was specifically higher in pituitary oncocytoma than in other subtypes of pituitary adenomas and in healthy pituitary glands (14). Therefore, in this study, we identified binding partners of 14-3-3η in pituitary oncocytoma by nanoLC-MS. A total of 1,195 proteins that bound either with 14-3-3η or with IgG were identified in pituitary oncocytoma. Of these proteins, 443 were identified as 14-3-3η binding proteins (Supplementary Table 2). Furthermore, 439 of 443 proteins were included in the 14-3-3-Pred database.

### Network Analysis of 14-3-3η Binding Proteins

**Figure 1:**
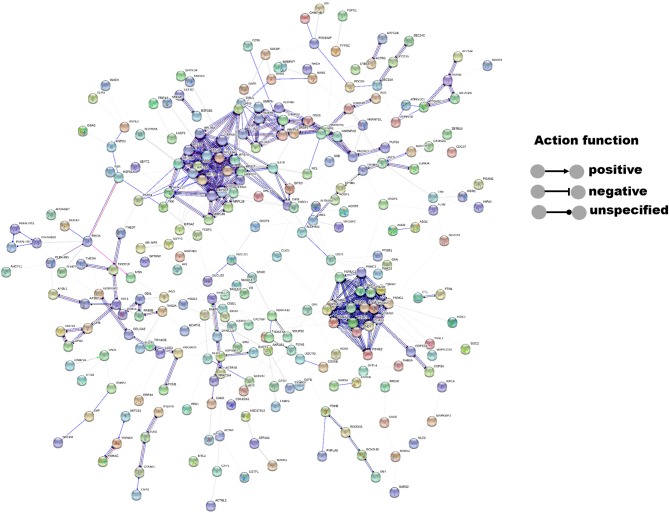
Interactions between 14-3-3η and its binding partners were determined by network analysis using the STRING database. Network nodes represent proteins, and edges represent protein–protein associations. The disconnected nodes were hidden in the network.

The interactions of 14-3-3η and its identified binding partners were determined by network analysis using the STRING database (Figure 1). The network consisted of 433 nodes (proteins) and 564 edges (protein–protein associations). The average node degree and average local clustering coefficient in the network were 2.61 and 0.325, respectively. The PPI enrichment *p*-value of the network was less than 1.0–16, indicating that proteins in the network exhibit more interactions among themselves than would be expected for a random set of proteins of similar size obtained from the genome. The PRAS40 (AKT1S1) protein was shown to exhibit experimental interactions with 14-3-3η. The combined score was 0.407, which suggests a functional link.

### Enrichment Analysis of 14-3-3η Binding Proteins

**Figure 2:**
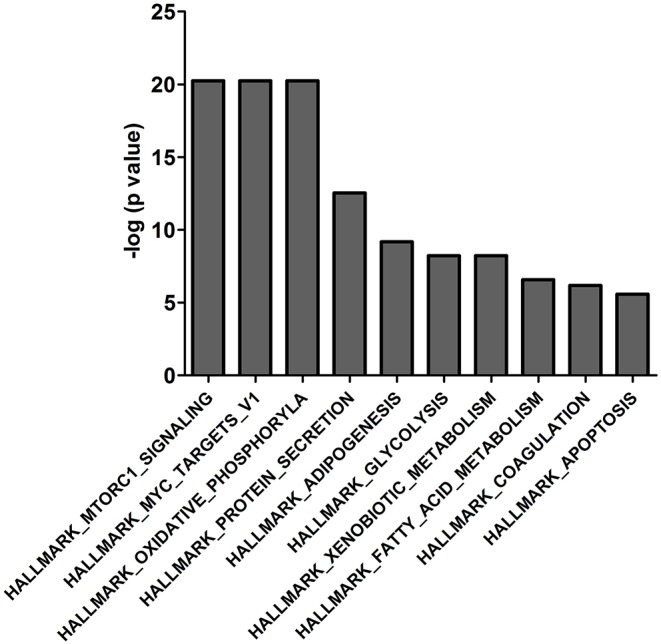
The top 10 enriched gene sets of the 439 binding proteins of 14-3-3η were determined based on the molecular signature database in the gene set enrichment analysis.

The 439 14-3-3η binding proteins were enriched in different signaling pathways according to the molecular signatures database used in the gene set enrichment analysis. The top 10 pathways are listed in Figure 2 and include HALLMARK_MTORC1_SIGNALING, HALLMARK_MYC_TARGETS_V1, HALLMARK_OXIDATIVE_PHOSPHORYLATION, HALLMARK_PROTEIN_ SECRETION, HALLMARK_ADIPOGENESIS, HALLMARK_GLYCOLYSIS, HALLMARK_XENOBIOTIC_METABOLISM, HALLMARK_FATTY_ACID_METABOLISM, HALLMARK_COAGULATION, and HALLMARK_APOPTOSIS. Interaction of PRAS40 (AKT1S1) with 14-3-3η was enriched in the PI3K_AKT_MTOR_SIGNALING pathway, as shown in Supplementary Table 3.

**Figure 3:**
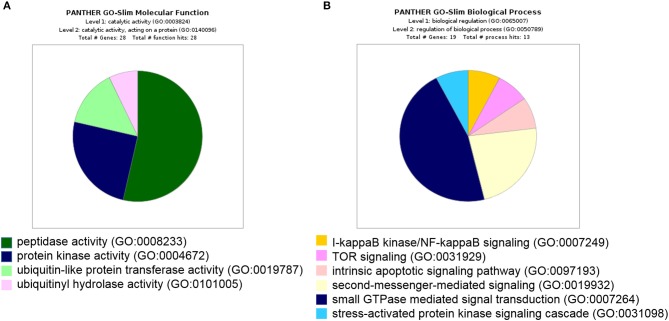
All 439 proteins that bound to 14-3-3η were classified based on molecular function and biological process in GO analysis. **(A)** The PRAS40 (AKT1S1) protein was categorized into protein kinase activity based on molecular function in GO analysis. **(B)** The PRAS40 (AKT1S1) protein was sorted into TOR signaling based on biological process in GO analysis.

Furthermore, all 439 proteins that bound to 14-3-3η were analyzed using the GO database. Based on the molecular function determined by GO analysis, the PRAS40 (AKT1S1) protein was classified in the protein kinase activity group (GO:0004672). As the binding partner of 14-3-3η, PRAS40 (AKT1S1) was enriched in TOR signaling (GO:0031929) (Figure 3).

Additionally, phosphorylation of substrates by Akt, including PRAS40, resulted in the subsequent binding of the substrate to 14-3-3η, which induced cell apoptosis and survival according to the 14-3-3 Induced Intracellular Signaling pathway described by QIAGEN (Supplementary Figure 1).

### Validation of PRAS40 Activation by Western Blot Analysis

**Figure 4:**
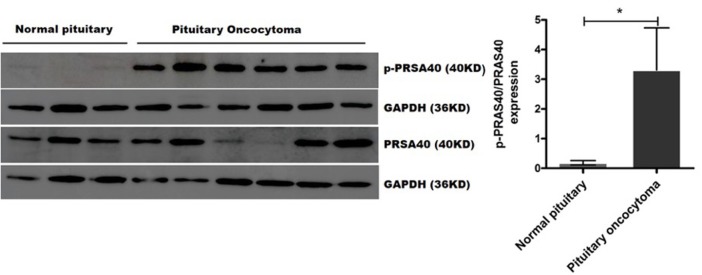
p-PRAS40 (T246) and PRAS40 protein expression between pituitary oncocytomas and normal pituitary glands by Western blot method. The relative expression of p-PRAS40 (T246)/PRAS40 in pituitary oncocytomas was significantly increased compared to normal pituitary glands. ^*^compared to control, *p* < 0.05.

We validated the expression of PRAS40, BAD, YAP1, and FOXO3A and their phosphorylated proteins (p-PRAS40 T246, p-BAD S112, p-YAP1 S127, and p-FOXO3A S253), which bind to 14-3-3η, in six pituitary oncocytoma and three healthy pituitary gland tissues by Western blot analysis. Notably, the expression of phosphorylated PRAS40 (T246) differed between pituitary oncocytomas and normal pituitary tissues. The relative expression of p-PRAS40 (T246)/PRAS40 was significantly higher in pituitary oncocytoma than in normal pituitary PNCAs (*p* = 0.0238; Figure 4). The expression of phosphorylated BAD (S112), YAP1 (S127), and FOXO3A (S253) did not differ between pituitary oncocytomas and normal pituitary tissues (Supplementary Figure 2).

### *In vitro* Experiments With a 14-3-3 Inhibitor

**Figure 5:**
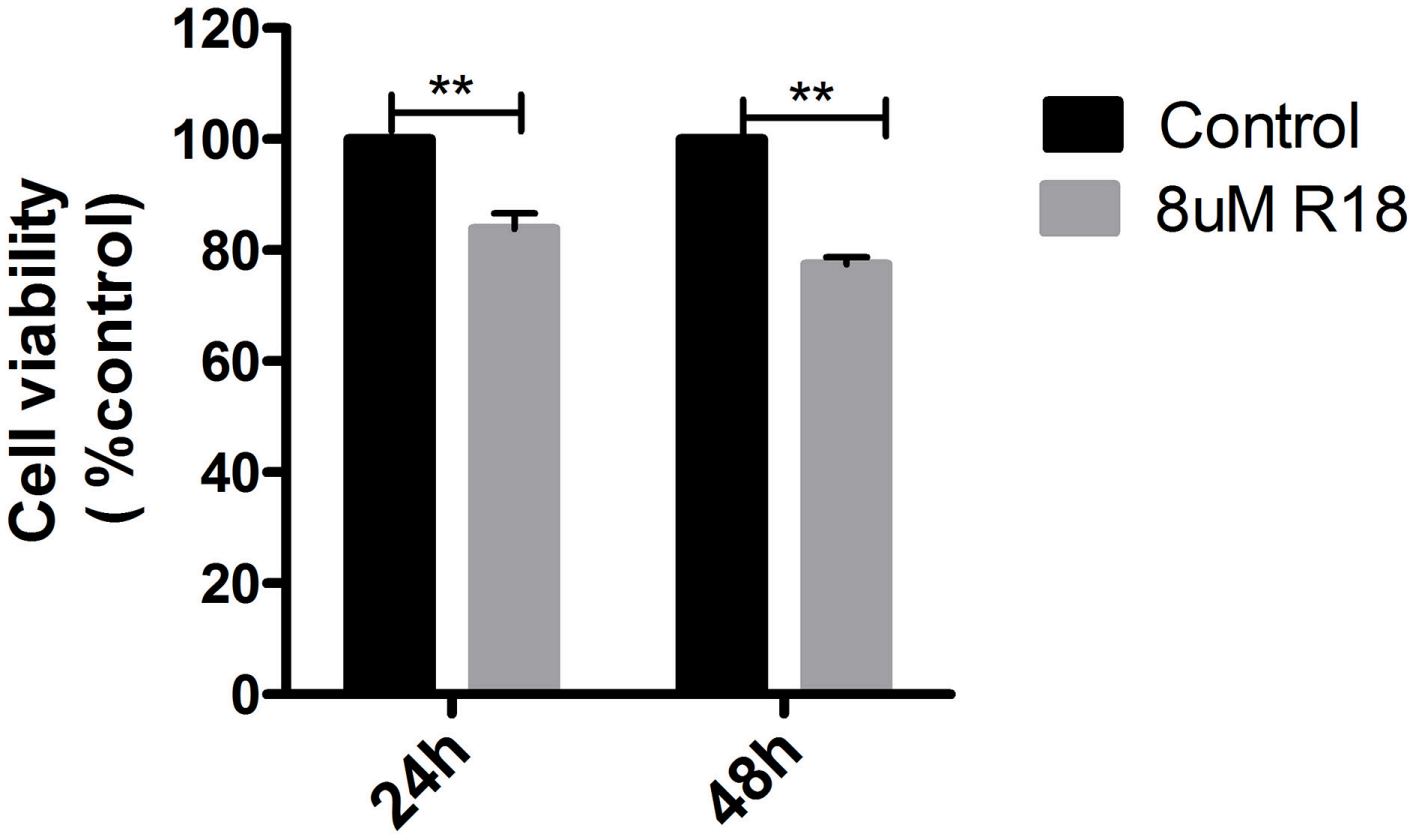
R18 suppresses tumor proliferation in pituitary oncocytoma. The cell viability was reduced to 83.8 ± 16.2 and 77.5 ± 22.5% after 24 and 48 h of 8 μm R18 in MMQ cells. All assays were performed in triplicate. ^**^Compared to control, *p* < 0.01.

R18, a 14-3-3 inhibitor, could inhibit cell proliferation in pituitary adenomas. MMQ cell lines were incubated with or without 8 μM R18 for 24 or 48 h before analysis with an MTS assay. The results demonstrated that R18 inhibited MMQ cell survival. The viability of MMQ cells was significantly decreased after treatment with 8 μM R18 for 24 and 48 h compared to the control group (*p* = 2.14 × 10^−8^ and 1.34 × 10^−5^; Figure 5).

## Discussion

The 14-3-3 proteins can bind to a multitude of cellular proteins, including kinases, phosphatases, and transmembrane receptors, which allows 14-3-3 to play important roles in a wide range of vital regulatory processes, such as neuronal development, mitogenic signal transduction, apoptotic cell death, cell cycle control, cell growth control, and viral and bacterial pathogenesis.

In our previous study, the expression of 14-3-3η protein was upregulated in pituitary oncocytoma, and the interaction of 14-3-3η with lactate dehydrogenase (LDHA) inhibited the metabolic pathway of glycolysis and enhanced mitochondrial biogenesis (14). In the present study, we aimed to identify the binding partners of 14-3-3η and their roles in the proliferation and survival of pituitary oncocytoma.

In this study, 443 binding partners of 14-3-3η were identified in pituitary oncocytoma by co-IP and nanoLC-MS analysis. PRAS40 was revealed to be an important binding partner of 14-3-3η by bioinformatics analysis based on experimental evidence. Furthermore, the upregulation of PRAS40 by phosphorylation at T246 was validated, which indicated that PRAS40 could be activated in pituitary oncocytoma. PRAS40 is reported to be a 14-3-3 binding protein based on its phosphorylation at T246 (16), which could be mediated by Akt, leading to its binding to 14-3-3η (17).

Additionally, 443 binding partners of 14-3-3η were identified by pathway enrichment analysis and GO analysis. PRAS40 is a crucial regulatory protein in the mammalian target of rapamycin mTOR complex 1 (mTORC1) signaling; it is an inhibitory component of mTORC1 (18, 19) and is responsible for regulating many functions of mTORC1 (20). Phosphorylation of T246 causes PRAS40 to dissociate from the mTORC1 complex and bind to 14-3-3 proteins (19). Therefore, phosphorylation of PRAS40 by Akt reduces its ability to inhibit mTORC1 (21). Studies have indicated that mTORC1 promotes the proliferation of tumor cells (22, 23). Thus, we speculated that phosphorylated PRAS40 (T246), after dissociation from the mTORC1 complex and binding to the 14-3-3η protein, promotes the proliferation of pituitary oncocytoma.

R18 is an inhibitor of 14-3-3 protein family members, and it binds to the conserved amphipathic groove in 14-3-3 proteins with high affinity (24). R18 potently eliminates interactions between 14-3-3 proteins and their target proteins and thus may abolish the ability of 14-3-3 proteins to inhibit cell proliferation. Previous studies elucidated that R18 inhibited the functions of 14-3-3 proteins in breast cancer cells, resulting in decreased proliferation (25). Unfortunately, no pituitary oncocytoma cell lines have been established. A previous study also indicated that prolactin upregulates the expression of 14-3-3ε in dairy cow mammary epithelial cells and that 14-3-3ε upregulates cell proliferation of dairy cow mammary epithelial cells via activating the PI3K-mTOR pathway (26). Thus, although pituitary oncocytoma cell lines were not available for this study, we identified the inhibitory effect of R18 on the proliferation of pituitary adenomas using the MMQ cell line. After 48 h of R18 treatment, cell proliferation was significantly decreased compared to that in the control group. This finding showed that R18 decreased cell proliferation in pituitary adenomas. The *in vitro* experiment showed that R18 was an effective inhibitor of 14-3-3η and may be used for the treatment of pituitary oncocytoma. We repeated the analyses with different concentrations of R18 in the MMQ cell line; the resulting dose-response curve is provided in the Supplementary Figure 3. The result showed that 8 μM of R18 is the most effective concentration in inhibiting the proliferation of the MMQ cell line. Higher concentrations did not further decrease the cell viability. Although R18 was effective in inhibiting cell proliferation, it was not a highly specific inhibitor for 14-3-3η due to its effect on other 14-3-3 proteins. Specific mRNA interference is a more effective method for validating the function of 14-3-3η in oncocytoma.

Regrettably, we did not detect BAD, FOXO3, or YAP1 expression in pituitary oncocytoma, which were phosphorylated substrates by Akt, in our experiment that combined co-IP with mass spectrometry. LC-MS/MS is a popular technique for detecting proteins in tissues. However, some peptides were present at low levels in samples and were thus difficult to detect via LC-MS/MS (27). Furthermore, peptide ions with very low signal levels are often difficult to distinguish from background noise (28). In summary, LC-MS/MS could not detect all of the proteins contained in tissues, which may be why we did not detect BAD, FOXO3, and YAP1. Although BAD, FOXO3, and YAP1 were not found, the interactions of the proteins PRAS40, BAD, FOXO3, and YAP1 with 14-3-3η play an important role in tumor apoptosis. Furthermore, BAD, FOXO3, and YAP1 expression were validated by Western blot. There were not significantly difference between normal pituitary and pituitary oncocytoma (unpublished data).

In conclusion, our data suggested a role of 14-3-3η in regulating the proliferation of pituitary oncocytoma. The elevated expression of 14-3-3η in pituitary oncocytoma could contribute to tumorigenesis. Moreover, the increased expression of p-PRAS40 (T246), which is a binding partner of 14-3-3η, indicated that 14-3-3η promoted cell proliferation by binding to PRAS40 (T246) and the activation of PRAS40. R18, an inhibitor of 14-3-3η, can reverse the effect of 14-3-3η and inhibit cell proliferation in pituitary oncocytoma. Further studies are required to determine the mechanism by which the 14-3-3η\PRAS40 (T246) complex promotes tumorigenesis.

## Data Availability Statement

All datasets generated for this study are included in the manuscript/Supplementary Material.

## Ethics Statement

The study has been approved by the appropriate ethics committee and was performed in accordance with the ethical standards laid down in the 1964 Declaration of Helsinki and its later amendments. The patients gave informed consent prior to their inclusion in this study.

## Author Contributions

JF and YZ conceived the idea and interpreted the data. JF, SZ, BL, and CL collected the samples, performed Co-IP and proteomic analyses. JF and HG performed the bioinformatic analysis. SZ, YM, YH, HW, LG, and DL established the cell model and performed *in vitro* experiments. JF and SZ aided in the data analysis and wrote the manuscript. All authors approved the submission.

### Conflict of Interest

The authors declare that the research was conducted in the absence of any commercial or financial relationships that could be construed as a potential conflict of interest.
