# Supplementary material for: The Apoptosis Regulator 14-3-3η and Its Potential as a Therapeutic Target in Pituitary Oncocytoma
Source: Front Endocrinol (Lausanne). 2019 Nov 28;10:797. doi: 10.3389/fendo.2019.00797 (PMC6893364; doi:10.3389/fendo.2019.00797)
Supplement: Supplementary file 4 [file Image_1.pdf]

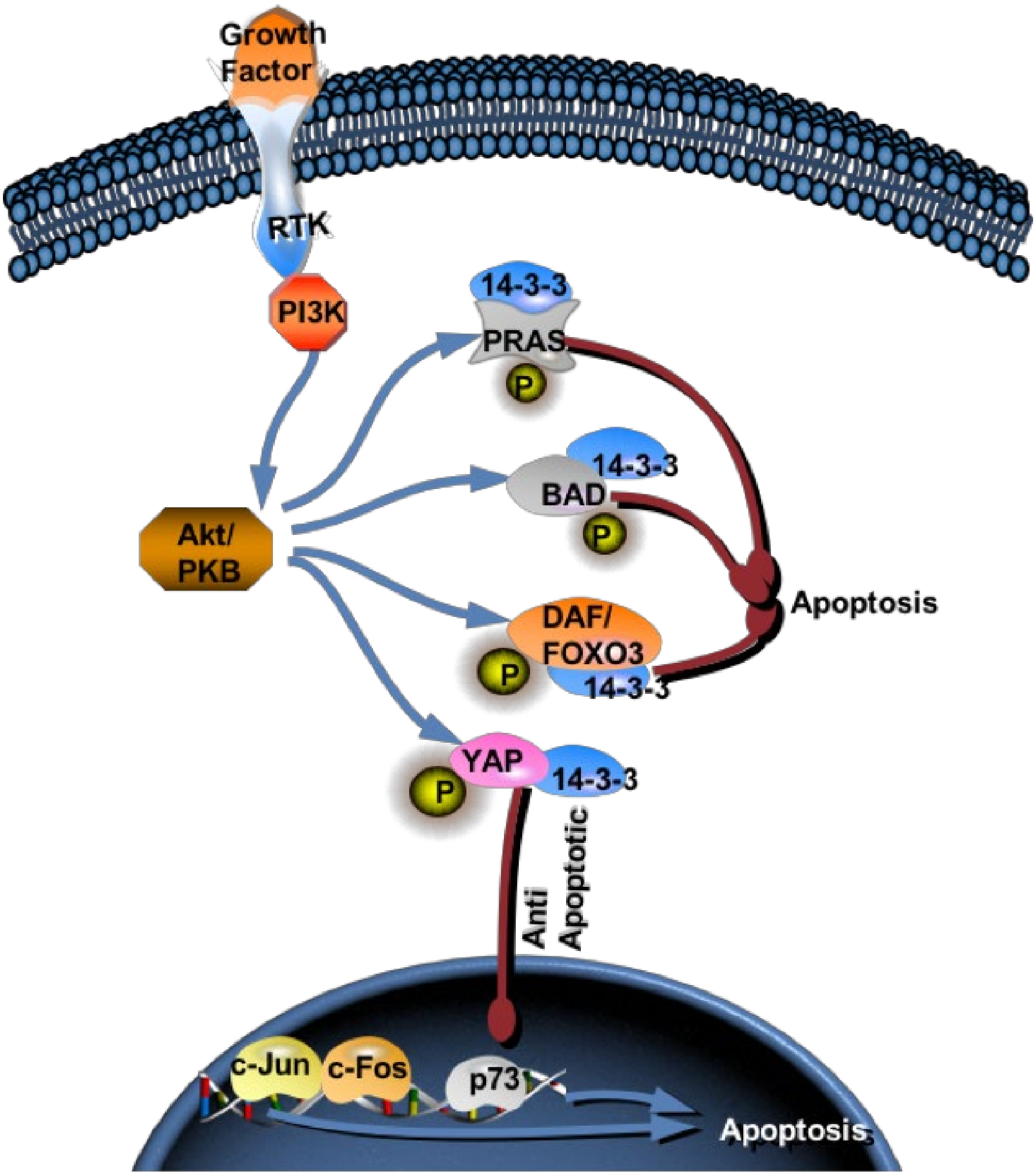

Supplementary Fig. 1 14-3-3 Induced Intracellular Signaling pathway described by QIAGEN. The phosphorylation of substrates by Akt, including PRAS40, resulted in the subsequent binding of the substrate to 14-3-3η.
