# Supplementary material for: The Apoptosis Regulator 14-3-3η and Its Potential as a Therapeutic Target in Pituitary Oncocytoma
Source: Front Endocrinol (Lausanne). 2019 Nov 28;10:797. doi: 10.3389/fendo.2019.00797 (PMC6893364; doi:10.3389/fendo.2019.00797)
Supplement: Supplementary file 5 [file Image_2.pdf]

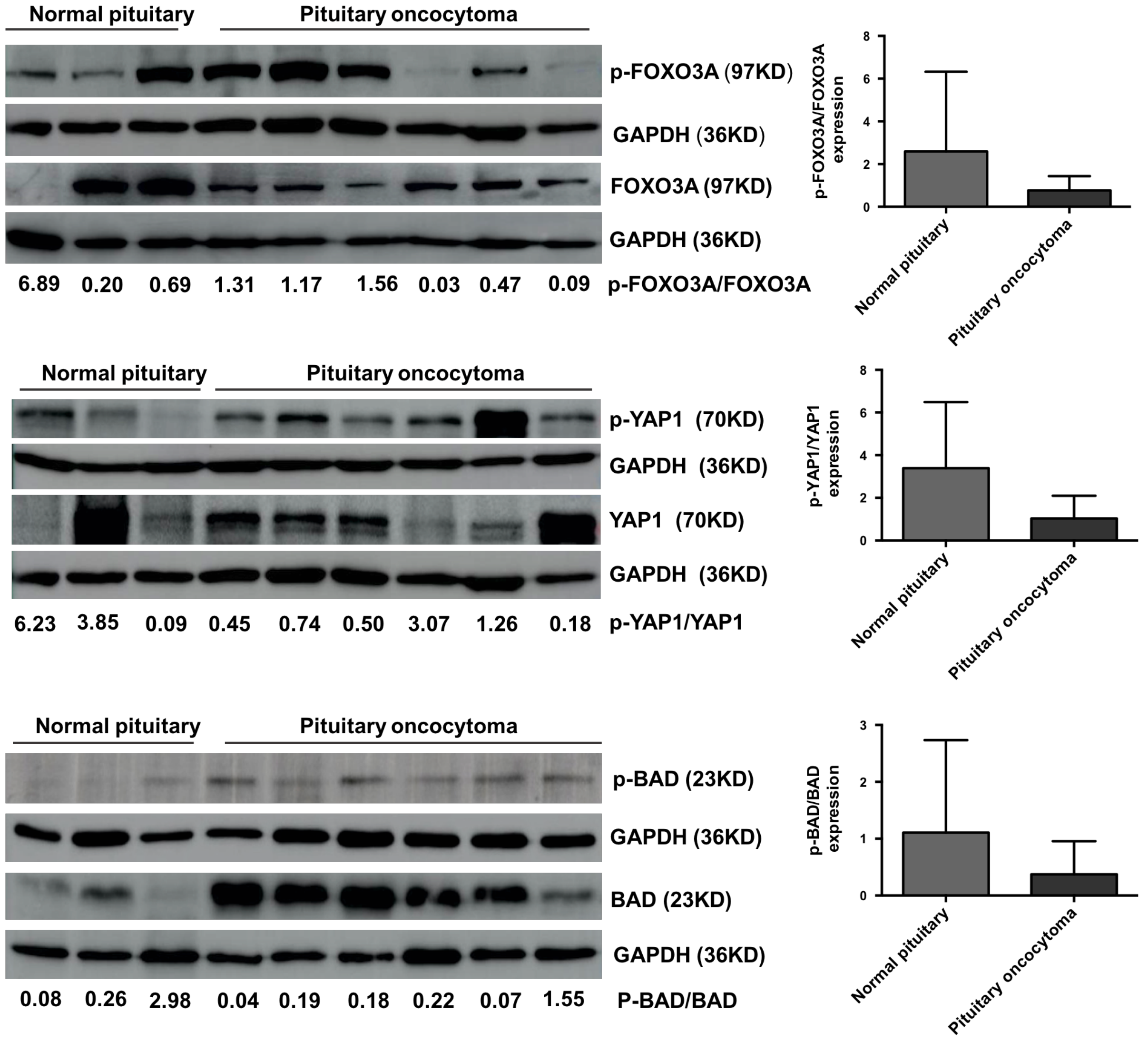

Supplementary Fig. 2 p-BAD S112, p-YAP1 S127, and p-FOXO3A S253 and BAD, FOXO3 and YAP1 protein expression between pituitary oncocytoma and normal pituitary glands by Western blot methods. There is no significant difference of phosphorylated BAD, FOXO3 and YAP1 between pituitary oncocytomas and normal pituitary gland.
