# Supplementary material for: The Apoptosis Regulator 14-3-3η and Its Potential as a Therapeutic Target in Pituitary Oncocytoma
Source: Front Endocrinol (Lausanne). 2019 Nov 28;10:797. doi: 10.3389/fendo.2019.00797 (PMC6893364; doi:10.3389/fendo.2019.00797)
Supplement: Supplementary file 6 [file Image_3.pdf]

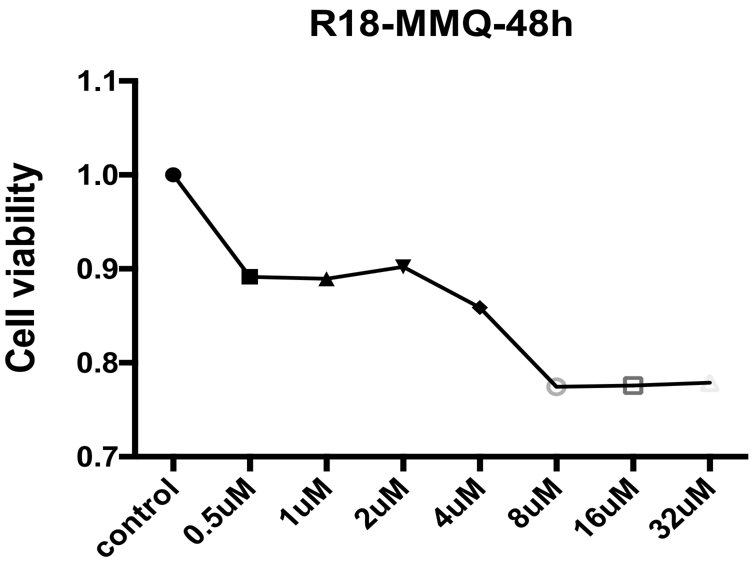

Supplementary Fig. 3 The analyses with different concentrations of R18 in MMQ cell line, and the dose-response curve. The result showed 8uM of R18 is the most effective concentration in inhibiting the proliferation of MMQ cell line. Higher concentration did not decrease the cell viability anymore.
